# Adjuvant chemotherapy for breast cancer after preoperative chemotherapy: A propensity score matched analysis

**DOI:** 10.1371/journal.pone.0234173

**Published:** 2020-06-05

**Authors:** Julie Labrosse, Marie Osdoit, Anne-Sophie Hamy, Florence Coussy, Jean-Yves Pierga, Fabien Reyal, Enora Laas

**Affiliations:** 1 Department of Surgery, Institut Curie, Paris, France; 2 Translational Research Department, Residual Tumor & Response to Treatment Laboratory, RT2Lab, INSERM, U932 Immunity and Cancer, Institut Curie, PSL Research University, Paris, France; 3 Department of Medical Oncology, Institut Curie, Paris, France; Mayo Clinic, UNITED STATES

## Abstract

Although identified to be at a higher risk of relapse, no consensus exists on the treatment of breast cancer (BC) patients with no pathological complete response after neoadjuvant chemotherapy (NAC). The benefit of adjuvant chemotherapy (ADJ) in this context has scarcely been studied. We evaluated the benefit of administrating adjuvant chemotherapy in a real life cohort of BC patients with invasive residual disease after NAC. 1199 female BC patients with T1-3NxM0 invasive tumors receiving NAC at Institut Curie from 2002 to 2012 were included in the analysis. 1061 had been treated by NAC only, whereas 138 had received additional adjuvant chemotherapy after NAC (FUN protocol: 5-FU-Vinorelbine). We compared disease-free survival (DFS) and overall survival (OS) rates between patients having received NAC only and patients having received NAC+ADJ. To ensure comparability of our populations, we used a propensity score (which defines the probability of treatment assignment conditional on observed baseline covariates) and matched each patient having received NAC+ADJ (n = 138) with a patient having received NAC only that had a similar propensity score value. Before propensity score matching, DFS and OS rates were significantly lower in the NAC+ADJ group compared to NAC only, after 3 years, 5 years and 10 years follow-up (p<0.01). After one-to-one PS matching, the two groups were comparable (n = 276 patients; 138 patients in each group). No significant difference was found regarding DFS (*p =* 0.87) or OS (*p =* 0.59) rates, neither in global population, nor by pathological subtype. Although our study did not show a benefit of administrating ADJ with FUN protocol (5-Florouracil- Vinorelbine) to BC patients with residual disease after NAC, further studies are warranted to determine the impact of other adjuvant regimens. Thereby, patients with little chance of responding to particular regimens could avoid the toxicity of futile therapy, and be study participants in evaluations of novel treatment strategies.

## Introduction

Neoadjuvant chemotherapy (NAC) is currently administered to patients with locally advanced breast cancers (BC), to BC of poor prognosis (triple negative and *HER2*- positive cancers, or BC with nodal involvement and/or high proliferation rates), or to early stage BC that have an indication of systemic therapy [1]. Beyond increasing breast-conserving surgery rates, it serves as an *in vivo* chemosensitivity test, facilitating early evaluation of the efficacy of systemic treatments, and making it theoretically possible to discontinue ineffective treatments [2–5]. Several staging systems were described to refine prognosis after NAC [6]. Furthermore, Cortazar *et al*. [7,8] showed that BC patients having reached pathological complete response (pCR) after NAC had higher disease-free survival (DFS) and overall survival (OS) rates, especially *HER2*-positive and triple negative breast cancers (TNBC).

However, no consensus exists on the treatment of patients who do not reach pCR after NAC, even though these patients are identified to be at a higher risk of relapse [9]. The benefit of administrating adjuvant chemotherapy (ADJ) in this context has scarcely been studied. Studies lead so far found discrepant results, with some randomized trials finding a trend towards better outcomes after ADJ [10,11], while others, attributing ADJ based on tumor characteristics, showed that it was associated to increased rates of distant metastasis and loco-regional recurrences [12]. Recently, ixabepilone randomly given to *HER2*-negative patients having residual invasive disease after standard anthracycline/taxane NAC regimen was not associated to higher recurrence-free survival nor overall survival rates [13]; similarly, zoledronate randomly administered to patients with residual disease after standard anthracycline/taxane NAC regimen did not show a benefit in terms DFS nor OS rates [14]. Conversely, the CREATE-X trial [15] randomly assigning 910 patients with residual disease after NAC to receive postsurgical treatment (radiotherapy +/- hormone therapy when indicated) either with or without Capecitabine showed that adjuvant Capecitabine significantly improved DFS and OS rates in *HER2*-negative BC. An explanation to these discrepancies relies in the fact that candidates to ADJ after NAC are of poorer prognosis from the start, making interpretation of the data difficult.

The objective of the current study was to evaluate the benefit of administrating adjuvant chemotherapy in a real life cohort of BC patients with invasive residual disease after NAC. To ensure comparability of our populations, we used a propensity score, which defines the probability of treatment assignment conditional on observed baseline covariates, and matched patients with similar PS values.

## Materials and methods

### Patients and tumors

Our cohort is a retrospective review of all patients receiving NAC at Institut Curie between 2002 and 2012. The cohort included 1199 female patients (NEOREP Cohort, CNIL declaration number 1547270), with T1-3/N0-3/M0 invasive, unifocal, unilateral, non-recurrent, non-metastatic BC, excluding T4 tumors (inflammatory, chest wall or skin invasion). The study was approved by the Institut Curie review board and ethics committee (Comité de Pilotage of the Groupe Sein), and was conducted according to institutional and ethical rules concerning research on tissue specimens and patients. In the French legal context, our institutional review board waived the need for written informed consent from the participants. All data were fully anonymized.

Information on clinical characteristics (age, menopausal status, body mass index) and tumor characteristics (tumor size, lymph node involvement, mitotic index, ki67, histological tumor grade, estrogen receptor (ER) status, progesterone receptor (PR) status, *HER2* status and histological response to NAC) were retrieved from medical health records.

Histological grade was described according to the Elston-Ellis modification of the Scarff-Bloom-Richardson grading system [16].

Hormone-receptor expression was analyzed by immunohistochemistry. Tumors were considered positive for ER or PR if 10% of carcinomatous cells displayed positive staining, as recommended by European guidelines [17]. *HER2* status was determined according to American Society of Clinical Oncology (ASCO) recommendations [18]. Based on immunohistochemistry surrogates, pathological BC subtypes were defined as follows: tumors positive for either ER or PR and negative for *HER2* were classified as luminal; tumors positive for *HER2* were considered *HER2*-positive BC; tumors negative for ER, PR, and *HER2* were considered triple negative BC (TNBC). Luminal BC were classified into luminal A or luminal B tumors, according to guidelines [19].

pCR was defined as the absence of residual invasive cancer cells in the breast and axillary lymph nodes (ypT0/is + / ypN0).

### Treatment protocol

Patients were treated according to national guidelines. NAC consisted in a sequential anthracycline–taxane regimen, with trastuzumab used in an adjuvant and/or neoadjuvant setting for *HER2*-positive tumors since the middle of the past decade. Trastuzumab treatments changed over time due to a change of marketing authorization during the study period. Patients with luminal BC received NAC in case of proliferative tumor. Surgery (breast-conserving or total mastectomy) was performed 4 to 6 weeks after NAC. Patients received adjuvant radiotherapy according to national guidelines. Indications of radiotherapy were: lumpectomy, total mastectomy in case of initial T3 or T4 tumors, all patients with involved axillary lymph nodes, and high-risk node-negative BC patients.

**Fig 1:**
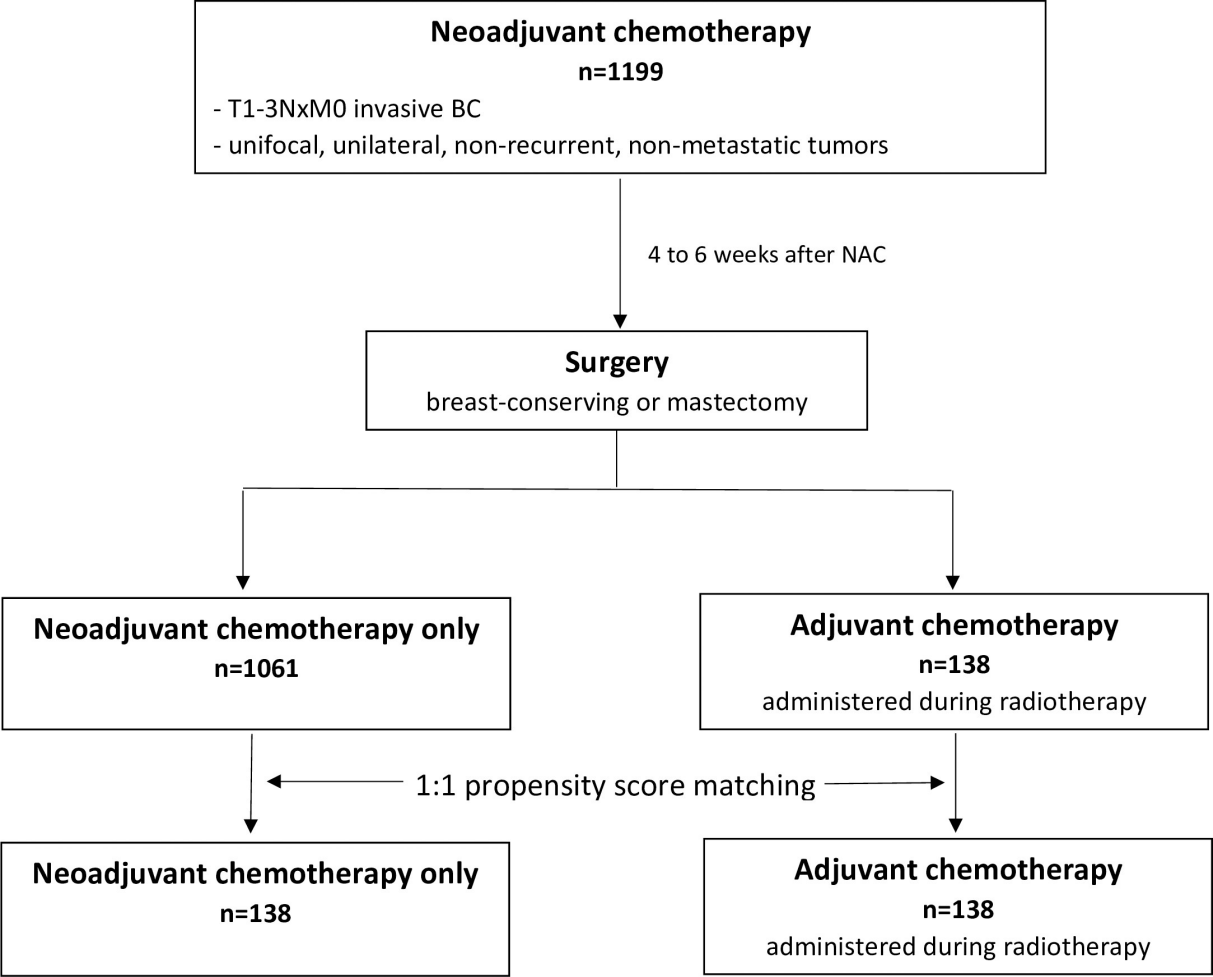
Flow chart.

After multidisciplinary consultation meeting, ADJ (FUN protocol: 5-Florouracil- Vinorelbine) was administered during radiotherapy to patients that had not reached pCR and/or with nodal involvement after NAC. Patients were treated with 5-FU, 500 mg/m2/d, over five consecutive days and vinorelbine,25 mg/m2 on days 1 and 6. Courses were repeated every 3 weeks for a total of four courses. Weekly dose intensity was defined, for all patients, as the total dose (mg/m2) divided by the total duration of the chemotherapy in weeks from day 1 of cycle one through one cycle length after the date of the last chemotherapy treatment.The choice of FUN protocol as ADJ regimen was based on the fact that vinorelbine is a potent inhibitor of mitotic polymerization that has radio-sensitizing effects, that can be administered during radiotherapy, and that is well tolerated by patients [20,21]. Adjuvant hormone therapy (tamoxifen, aromatase inhibitor, or GnRH agonists) was prescribed when indicated to patients with luminal disease, sequentially to radiotherapy (and sequentially to ADJ when administered). Patient follow-up after treatment was of every 3 months during the first 2 years, then every 6 months during 3 years, and once a year starting from the 5th year. Follow-up consisted of clinical examination associated to mammography and mammary ultrasound once a year (Fig 1).

### Study endpoints

**DFS** was defined as the time from surgery until the date of: recurrence of ipsilateral breast or loco-regional tumor, distant recurrence, or death from any cause, whichever occurred first.

**OS** was defined as the time from surgery to death or to last follow-up date in absence of death. Patients for whom none of these events were recorded were censored at the date of their last known contact.

### Statistical analysis

Missing data were handled by multiple imputations by chained equations.

Since patients were not randomly allocated to ADJ or no ADJ, a propensity score (PS) was built and used to control for selection bias. Propensity score analysis, a post hoc adjustment method, consists in deriving the conditional probability of receiving the treatment for a patient given observed covariates. Matching each treated patient to an untreated one who has the most similar PS tends to balance baseline characteristics between the two groups, thus reducing the risk for overt bias given observed covariates [22–27].

**Fig 2:**
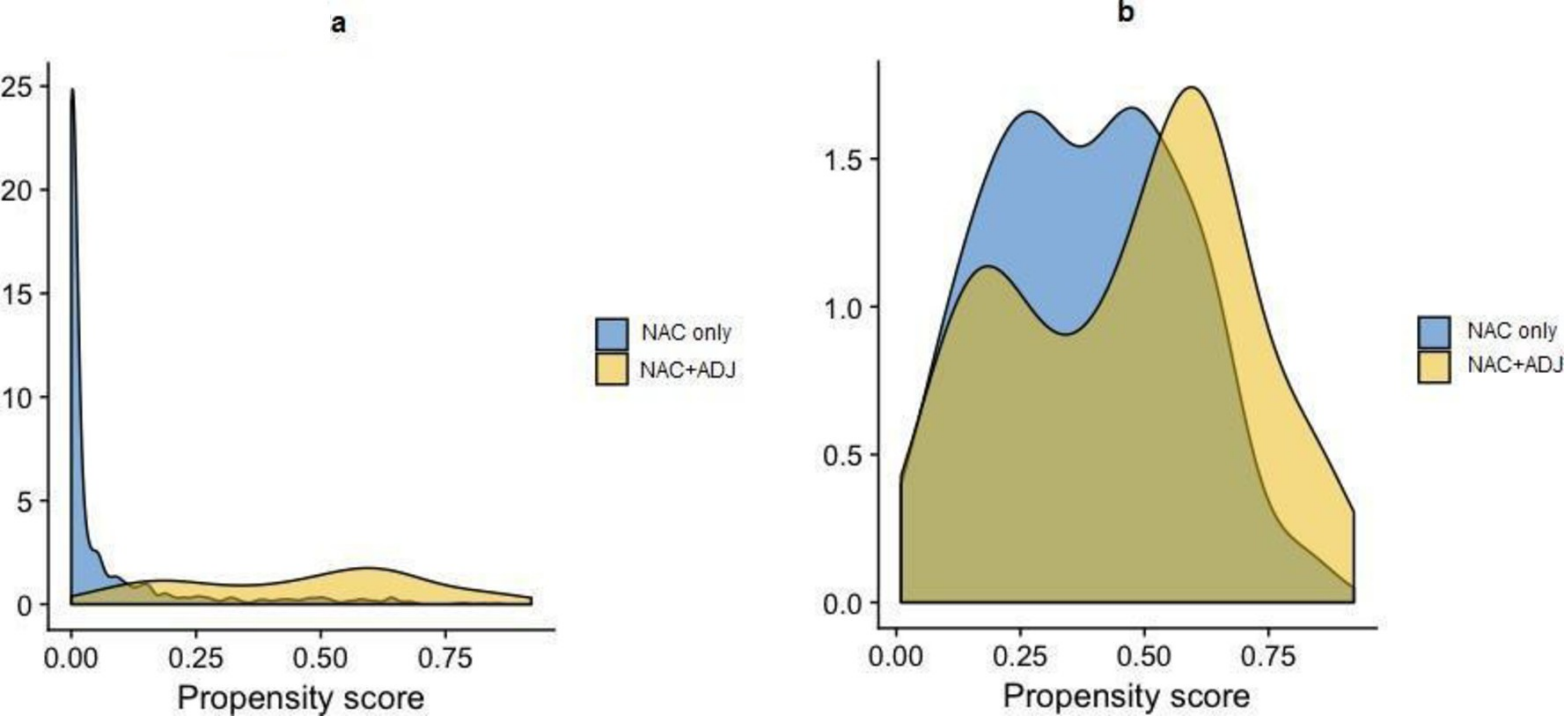
a-b. Propensity score distribution in the NAC only and NAC+ADJ groups, before (Fig 2a) and after (Fig 2b) PS matching.

Univariate analysis was used to compare baseline patient characteristics for both groups. We used a multivariate logistic regression model to generate PS in order to match patients who received ADJ with patients who did not receive ADJ. Covariates included in the model were patient characteristics (age, menopausal status, body mass index), histological characteristics (pre-operatory tumor size, histological type and grade, mitotic index), type of breast surgery, type of axillary surgery, number of involved nodes, margin status, histological response status, and post-operatory node status. We performed a 1-to-1 matching by PS to the nearest neighbor method, with a caliper width equal to 0.25 standard deviations, without replacement. PS in the original treated and control groups and in the matched treated and control groups are described Fig 2A and 2B. The benefit of ADJ was estimated by directly comparing outcomes between patients with or without ADJ in the matched sample (with paired-tests).

Analyses were performed with R software, version 3.3. Qualitative variables were compared by Chi-Square or Fisher Exact tests and quantitative ones by Student T-tests. Survival probabilities were estimated by Kaplan-Meier method, and survival curves were compared with Log-Rank tests. Hazard Ratios (HR) and their 95% Confidence Intervals (CI) were calculated with the Cox Proportional Hazards model. Significance threshold was of 5%.

## Results

### Analyses before PS matching

Our cohort was composed of 1199 BC patients, of which 1061 patients treated with NAC only and 138 patients treated with NAC+ADJ (FUN protocol) (Fig 1). Before PS matching, patient characteristics according to adjuvant treatment status are described Table 1.

**Table 1:** Patient characteristics in the NAC only and NAC+ADJ groups, before PS matching.

	NAC only (n = 1061)	NAC+ADJ (n = 138)	*p*-value
**Age** [median (+/- SD)]	48.73 (+/- 10.1)	47.01 (+/- 9.63)	0.06
≥ 40 years old	831 (78.5%)	103 (74.6%)	0.31
< 40 years old	228 (21.5%)	35 (25.4%)	
**Body Mass Index** [median (+/- SD)]	24.74 (+/- 4.76)	24.75 (+/- 4.09)	0.98
< 20	129 (12.2%)	12 (8.7%)	0.21
20–30	783 (74.2%)	112 (81.2%)	
>30	143 (13.6%)	14 (10.1%)	
**Menopausal**			0.42
No	656 (62.4%)	91 (65.9%)	
Yes	395 (37.6%)	47 (34.1%)	
**Histology**			0.7
Ductal	935 (89.7%)	127 (92.0%)	
Lobular	67 (6.4%)	7 (5.1%)	
Other	40 (3.8%)	4 (2.9%)	
**Immunohistochemistry Subtype**			0.0036
Luminal A	294 (27.7%)	50 (36.2%)	
Luminal B	157 (14.8%)	29 (21.0%)	
TNBC	339 (32.0%)	36 (26.1%)	
*HER2*-positive	271 (25.5%)	23 (16.7%)	
**Grade Elston Ellis**			0.12
1	45 (4.4%)	2 (1.5%)	
2	375 (36.5%)	57 (43.5%)	
3	606 (59.1%)	72 (55.0%)	
**Mitotic Index** [median (+/- DS)]	20.94 (+/- 19.2)	19.29 (+/- 19.2))	0.36
0–10	321 (33.7%)	50 (38.8%)	0.46
11–22	292 (30.6%)	39 (30.2%)	
> 22	340 (35.7%)	40 (31.0%)	
NA*	106	9	
**Tumor size**			0.25
Clinical [average(SD)]	45.13 (+/- 19.5)	47.2 (+/- 21.8)	0.87
≤ 20 mm	81 (7.6%)	10 (7.2%)	
> 20 mm	979 (92.4%)	128 (92.8%)	
**Baseline TN Stage**	60 (5.7%)	10 (7.2%)	0.66
T1			
T2	710 (66.9%)	88 (63.8%)	
T3/T4	291 (27.4%)	40 (29.0%)	
N0	499 (47.0%)	26 (19.0%)	<0.0001
N1	519 (48.9%)	101 (73.7%)	
N2/3	43 (4.1%)	10 (7.2%)	
**Therapeutic Treatment**			
Neoadjuvant Trastuzumab	189 (17.8%)	16 (11.6%)	0.068
Breast-conserving surgery	722 (68.2%)	75 (54.3%)	0.0012
Total mastectomy	337 (31.8%)	63 (45.7%)	
**Residual disease**			
No pCR	768 (72.5%)	137 (99.3%)	<0.0001
Tumor size [median(+/-SD)]	17.77 (+/- 17.5)	27.79 (+/- 21.3)	<0.0001
**Number of nodes withdrawn**	11.59 (+/- 4.95)	12.12 (+/- 4.19)	0.13
**Positive node**	526 (50.0%)	131 (94.9%)	<0.0001
**Adjuvant Treatment**	1059 (100%)	138 (100%)	<0.0001
Radiotherapy			
Hormonal Therapy	559 (53.6%)	94 (68.1%)	0.0013
Trastuzumab	235 (22.1%)	23 (16.7%)	<0.0001
**NAC protocol**			<0.0001
Anthracycline based regimens	234 (22.0%)	1 (0.7%)	
Taxane based regimens	24 (2.3%)	1 (0.7%)	
Anthracycline-taxane regimens	709 (66.8%)	136 (98.6%)	
Other	94 (8.9%)	0 (0.0%)	

For most patients in the NAC only (n = 1061) and NAC+ADJ (n = 138) groups, **NAC** consisted in a sequential treatment by anthracyclines followed by taxanes. All patients receiving **ADJ** were treated by 5-Fluorouracil–Vinorelbine (FUN protocol).

Several patterns were significantly different according to adjuvant treatment status. Histological subtypes were significantly different between the two groups, with more luminal tumors in the NAC+ADJ group (57.2% *vs*. 42.5%, respectively, *p =* 0.0036). The NAC+ADJ group was associated to higher nodal involvement rates (N1: 73.7% for NAC+ADJ *vs*. 48.9% for NAC only, *p*<0.0001). 45.7% of NAC+ADJ patients had total mastectomy *vs*. 31.8% in the NAC only group (*p =* 0.0012). After breast-conserving surgery, larger residual tumor size (*p*<0.0001) and more nodal involvement (*p*<0.0001) were observed in NAC+ADJ patients compared to NAC only patients (Table 1).

**Fig 3:**
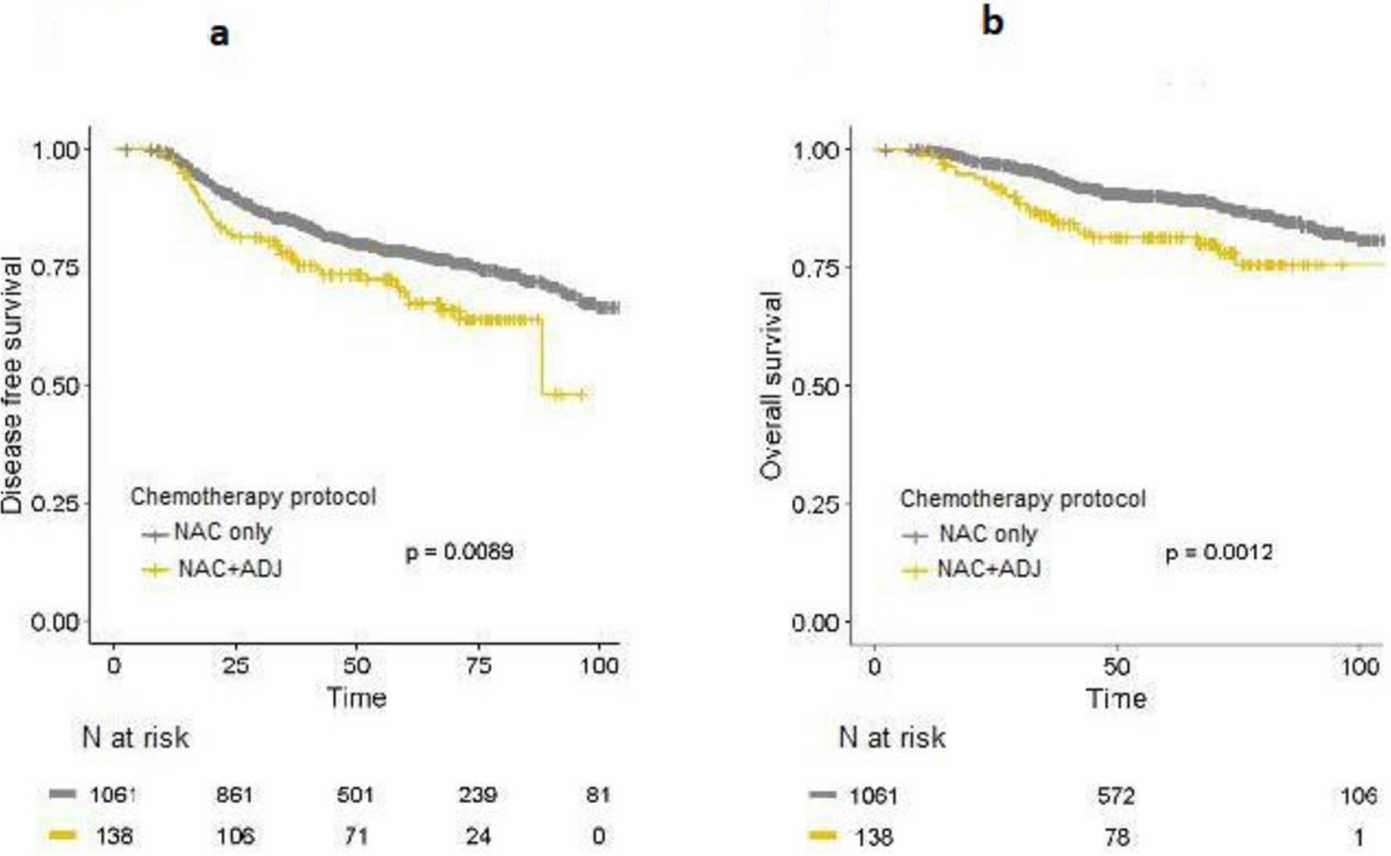
a-b. DFS (Fig 3a) and OS (Fig 3b) curves for whole population in the NAC only and NAC+ADJ groups, before PS matching.

Before PS matching, DFS (HR = 1.55; 95%IC [1.11–2.15]; *p =* 0.0092) and OS (HR = 2.01; 95%IC [1.3–3.08]; *p =* 0.0012) rates were significantly lower in the NAC+ADJ group compared to the NAC only group, after 3years, 5 years and 10 years of follow-up (Fig 3A and 3B).

### Analyses after PS matching

We formed matched sets of patients with similar PS values (one-to-one matching), excluding those with no matched PS. Our final population study was composed of 276 patients (138 in each group). The two groups were comparable after PS matching (Fig 2A and 2B).

Patient characteristics are described Table 2. No pattern was significantly different between the NAC and NAC+ADJ groups regarding age, menopausal status, tumor size and type, mitotic index, node involvement, nor in terms of breast-conserving surgery and pCR rates.

**Table 2:** Patient characteristics in the NAC only group and NAC+ADJ groups, after PS matching.

	NAC only (n = 138)	NAC+ADJ (n = 138)	*p*-value
**Age** [median]	49.3	46.1	0.06
≥ 40 years old	114 (82.6%)	103 (74.6%)	0.11
< 40 years old	24 (17.4%)	35 (25.4%)	
**Body Mass Index** [median (+/- SD)]	24.57 (+/- 4.58)	24.75 (+/- 4.09)	0.73
< 20	15 (10.9%)	12 (8.7%)	0.75
20–30	107 (77.5%)	112 (81.2%)	
>30	16 (11.6%)	14 (10.1%)	
**Menopausal**			0.71
No	88 (63.8%)	91 (65.9%)	
Yes	50 (36.2%)	47 (34.1%)	
**Histology**			0.7
Ductal	123 (89.1%)	127 (92.0%)	
Lobular	9 (6.5%)	7 (5.1%)	
Other	6 (4.3%)	4 (2.9%)	
**Immunohistochemistry Subtype**			0.88
Luminal A	39 (28.2%)	50 (36.2%)	
Luminal B	43 (31.2%)	29 (21.0%)	
TNBC	36 (26.1%)	36 (26.1%)	
*HER2*-positive	20 (14.5%)	23 (16.7%)	
**Grade Elston Ellis**			0.64
1	5 (3.6%)	3 (2.2%)	
2	65 (47.1%)	61 (44.2%)	
3	68 (49.3%)	74 (53.6%)	
**Mitotic Index** [median (+/- DS)]	16.08 (+/- 16.6)	18.94 (+/- 18.7)	.18
0–10	56 (40.6%)	54 (39.1%)	0.35
11–22	51 (37.0%)	43 (31.2%)	
> 22	31 (22.5%)	41 (29.7%)	
**Tumor size**			
Clinical [average(SD)]	48.82 (+/- 20.6)	47.2 (+/- 21.8)	0.53
≤ 20 mm	10 (7.2%)	10 (7.2%)	0.99
> 20 mm	128 (92.8%)	128 (92.8%)	
**Baseline TN Stage**			
T1			0.35
T2	87 (63.0%)	88 (63.8%)	
	46 (33.3%)	40 (29.0%)	
T3/T4			0.069
N0	41 (29.7%)	26 (18.8%)	
N1	91 (65.9%)	101 (73.2%)	
N2/3	6 (4.3%)	11 (8.0%)	
**Therapeutic Treatment**			
Neoadjuvant Trastuzumab	189 (17.8%)	16 (11.6%)	0.22
Breast-conserving surgery	71 (51.4%)	75 (54.3%)	0.63
Total mastectomy	67 (48.6%)	63 (45.7%)	
**Residual Disease**			
No pCR	138 (100%)	137 (99.3%)	0.99
Tumor size [median(+/-SD)]	25.85 (+/- 19.2)	28.11 (+/- 21.5)	0.36
**Number of nodes withdrawn**	12.07 (+/- 5.23)	12.12 (+/- 4.19)	0.94
**Positive node**	135 (97.8%)	131 (94.9%)	0.2
**Adjuvant Treatment**			
Radiotherapy	138 (100%)	138 (100%)	<0.0001
Hormonal Therapy	90 (65.2%)	94 (68.1%)	0.61
Trastuzumab	10 (7.2%)	16 (11.5%)	0.3
**NAC protocol**			
Anthracycline based regimens	0 (0.0%)	1 (0.7%)	0.06
Taxane based regimens	1 (0.7%)	1 (0.7%)	
Anthracycline-taxane regimens	132 (95.7%)	136 (98.6%)	
Other	5 (3.6%)	0 (0.0%)	

**Fig 4:**
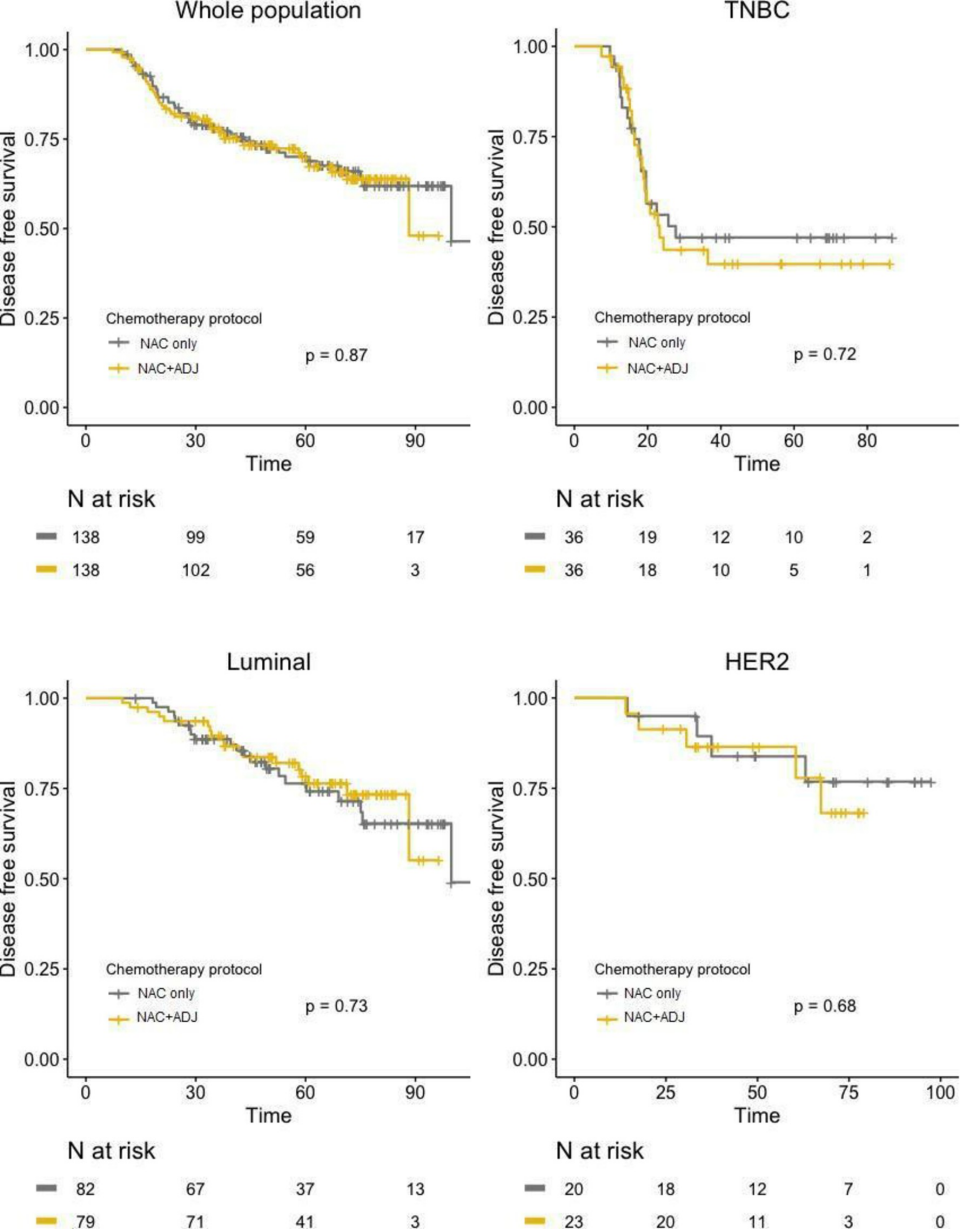
DFS curves in NAC only and NAC+ADJ groups for whole population and by pathological subtype (TNBC, luminal, and HER2-positive tumors), after PS matching.

**Fig 5:**
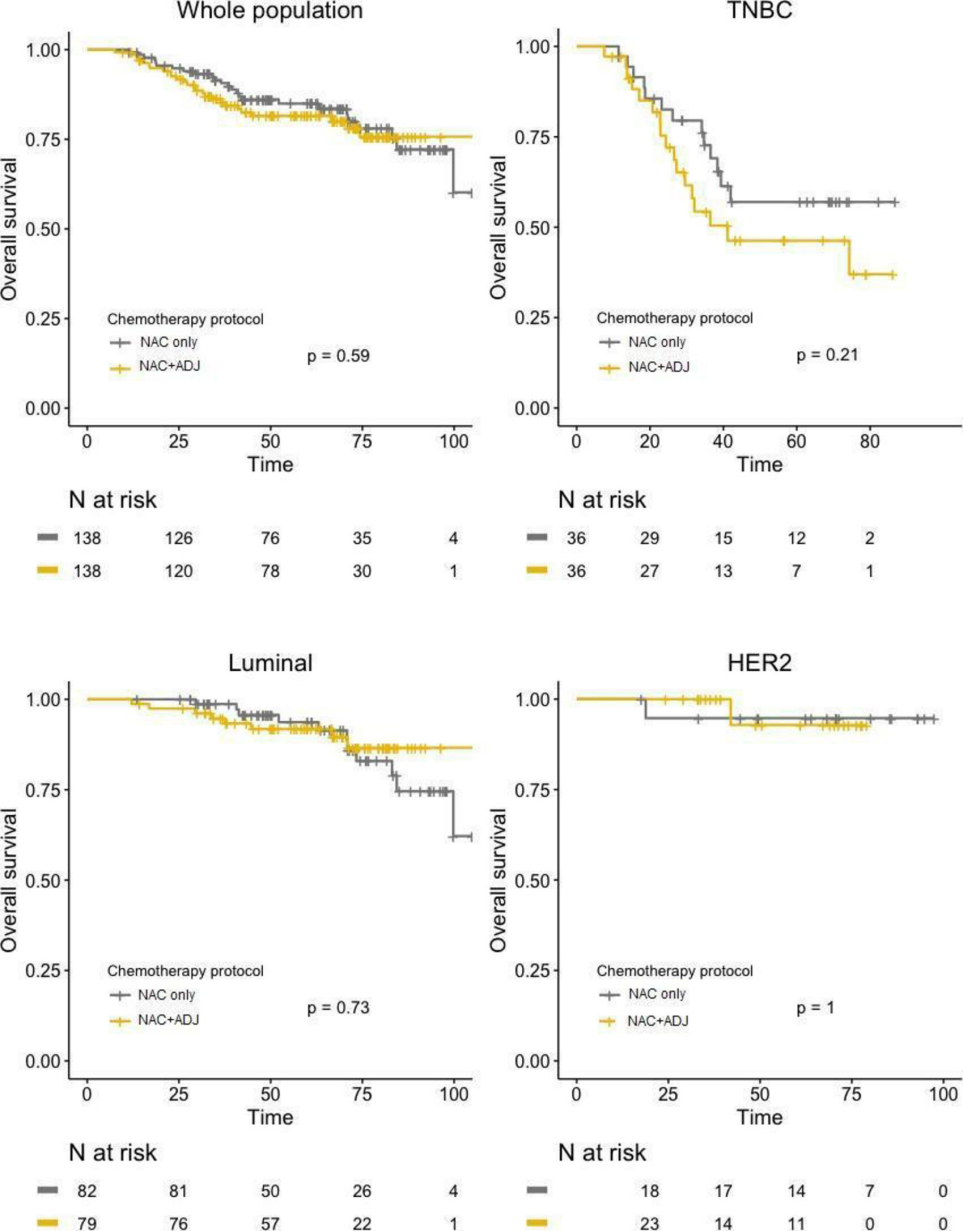
OS curves in NAC only and NAC+ADJ groups for whole population and by pathological subtype (TNBC, luminal, and HER2-positive tumors), after PS matching.

After PS matching, no significant difference was found between the two groups regarding DFS, neither in global population (HR = 1.04; 95%IC [0.67–1.59]; *p =* 0.87), nor after stratification by pathological BC subtype (Fig 4). Similar results were observed for OS, with no significant difference in whole population (HR = 1.17; 95%IC [0.67–2.03]; *p =* 0.59), nor in any of the BC subtypes (Fig 5).

## Discussion

In our PS matched analysis, administrating ADJ with FUN protocol (5-Fluorouracil–Vinorelbine) to patients with residual disease after NAC showed no benefit in terms of disease-free survival nor overall survival outcomes over patients treated with NAC alone. Similar results were observed after stratification by pathological BC subtype, as adjunction of ADJ after NAC did not significantly affect prognosis neither for luminal, nor for TNBC, nor *HER2*-positive tumors.

Our results are coherent with the major studies exploring the benefit of ADJ in patients with residual disease after NAC. Thomas *et al*. [10] were the first to evaluate the use of an alternate ADJ regimen in women with no pCR after NAC (Vincristine, Doxorubicin, Cyclophosphamide, Prednisone). In the adjuvant setting, some patients (n = 51) randomly received the same treatment as NAC, while others (n = 55) received a different regimen (VbMF: Vinblastine, Methotrexate with calcium leucovorin rescue, and Fluorouracil). Although a trend towards changing regimen was observed, results were not significant in terms of DFS and OS after a median follow-up of 13.9 years (p = 0.16). Likewise, in the NSABP-B27 study [11], women with operable BC (n = 2,411) were randomly assigned to receive preoperative doxorubicin and cyclophosphamide followed by surgery, doxorubicin and cyclophosphamide followed by docetaxel and then surgery, or doxorubicin and cyclophosphamide followed by surgery and then by adjuvant docetaxel. OS and DFS rates were not significantly higher for patients receiving ADJ (p = 0.51 for OS and p = 0.24 for DFS, respectively).

More recently, *HER2*-negative invasive BC patients with residual disease after standard anthracycline/taxane NAC regimen were randomized either to 6 cycles of adjuvant ixabepilone (n = 19) or observation (n = 24) [13]. The 3-year recurrence-free survival and OS rates were not statistically different between the two groups (p = 0.35 and p = 0.18, respectively), but the study was closed early due to significant toxicities in the ixabepilone arm. Similarly, patients with invasive residual tumor after a minimum of four cycles of anthracycline-taxane NAC regimen were randomized within 3 years after surgery to receive zoledronate for 5 years versus observation. After a median time of 54.7 months, no difference in DFS nor OS rates was observed between the zoledronate and observation groups (*p* = 0.789 and *p* = 0.408, respectively) [14].

Surprisingly, Knauer *et al*. [28] found that extended chemotherapy was associated to poorer survival outcomes. Patients were either treated by NAC only (Epirubicin and Docetaxel, n = 45), ADJ (Epirubicin, Cyclophosphamide +/- Taxane, n = 221), or NAC+ADJ (n = 90). Extended chemotherapy resulted in a significantly reduced 5-year DFS (61% for NAC+ADJ *vs*. 85% for NAC only and 82% for ADJ only; *p =* 0.008) and increased rates of distant metastasis and loco-regional recurrences (*p =* 0.033). However, the fact that attributing adjuvant treatment was based on tumor characteristics may have introduced bias, as the NAC+ADJ group comprised tumors of poorer prognosis.

Conversely, discrepant results were observed in the CREATE-X trial [15,29], in which 910 patients with *HER2*-negative residual invasive disease after NAC (anthracycline and taxane based regimens) were randomly assigned to postsurgical treatment (radiotherapy +/- hormone therapy when indicated) either with or without Capecitabine. DFS and OS rates were significantly higher in the Capecitabine group (5year-DFS: 74.1% *vs*. 67.7% for control group, respectively, *p* = 0.01 and OS: 89.2% *vs*. 83.9% for control group, respectively, *p =* 0.01). Concerning subtypes, the strongest benefit was seen in TNBC (which represented 30% of cases), with DFS rates of 69.8% in the Capecitabine group *vs*. 56.1% in the control group (HR 0.58; 95% CI [0.39–0.87]), and OS rates of 78.8% *vs*. 70.3%, respectively (HR 0.52; 95% CI [0.30–0.90]). Notably, results for hormone receptor positive tumors were of less magnitude (HR = 0.81 for DFS; HR = 0.73 for OS). The study was lead on Japanese patients, which should be considered in the interpretation of these data, as their metabolism may differ from that of occidental patients. These results are not consistent with the CIBOMA study which looked at the effect of adjuvant Capecitabine on TNBC. Results of CIBOMA did not conclude to increased survival rates in the overall TNBC population but reported a non-significant difference for patients with non-basal tumors.

Results of our analysis need to be interpreted in the context of potential limitations. The small effectives and the retrospective character of our study may lead to potential bias. After adjustment on the PS, our population became smaller with 276 patients. We decided to follow the statistical recommendation of using the PS to gain its statistical power despite the decreasing of our cohort. Although patients included were treated for BC from 2002 to 2012, the treatment consisted in anthracycline-based regimens or sequential anthracycline–taxane regimens, which is coherent with the standard current practices. The adjuvant regimen was composed of FUN. In current practice, it is no longer used but at the time period analyzed in this study (2002–2012), this regimen was commonly used. As it is currently acknowledged that chemotherapy is less efficient in luminal tumors [30], our results could be influenced by the important representation of luminal tumors in our NAC+ADJ cohort. The NAC+ADJ group was mostly composed of luminal tumors (57.2% *vs*. 26.1% for TNBC and 16.7% for *HER2*-positive, *p =* 0.88), which could have attenuated the ability to demonstrate the benefit of ADJ. The impact of hormone therapy in luminal tumors should also be considered, as it may be difficult to discriminate between the advantage obtained with chemotherapy and that related to endocrine treatment. Hormone therapy was given to 68.1% patients in the NAC+ADJ group *vs*. 65.2% patients NAC only group (*p =* 0.61). ADJ significantly delayed initiation of hormone therapy, as hormone therapy was started 24 days later (median time from surgery to initiation of hormone therapy: 99 days for NAC only group *vs*. 123 days for NAC+ADJ group, respectively, p<0.001). However, the risk associated to each day passed without hormone therapy was not significant (HR = 0.96, *p =* 0.69).

Ensuring comparability between populations is a major challenge in studies analyzing the benefit of ADJ after NAC, tumors in the groups treated by ADJ being of poorer prognosis from the start. Randomization is in practice difficult to set up since it implies not proposing any additional chemotherapy treatment to patients with poor prognosis tumors. In our study, precise identification of characteristics taken into account to attribute ADJ enabled to build a regression model, from which probability of receiving ADJ was then calculated. The effectiveness of PS matching has already been demonstrated, as stratification on the estimated PS in studies lead so far consistently reduced systematic baseline differences [25–27]. Populations compared were relatively different from the start; hence, a caliper width of 0.25SD was chosen to make the two groups comparable by using PS matching. A smaller caliper width would have eliminated too many patients from the analysis. After one-to-one PS matching, the two populations were therefore comparable; their only difference was presence or absence of ADJ after NAC. Still, PS matching cannot be considered equal to randomization. Whereas randomization randomly distributes variables, covariates used to define PS were those recorded in our database and are therefore restricted. Patients with no matched PS value were excluded, thus reducing the power of our study. However, recent simulation studies have demonstrated that matching or weighting on PS were the optimal methods to control systematic differences between groups.”

In a time when treatment for breast cancer is individualized, it seems that the choice of adjuvant treatment in patients with residual disease after NAC should also be personalized and targeted to pathological subtypes. Although our study did not show a benefit of the FUN protocol (5-Florouracil- Vinorelbine) in this setting, the benefit of other adjuvant regimens cannot be excluded. Further studies are expected to confirm Capecitabine’s impact [31]; the CIBOMA randomized trial is currently in progress to evaluate the efficacy of adjuvant Capecitabine after standard chemotherapy in TNBC [32]. Concerning HER2-positive tumors, major pCR and DFS gains were observed since the trastuzumab and pertuzumab era. The Katherine Clinical Trial evaluated the efficacy and safety of T-DM1 (trastuzumab, covalently linked through a molecular bond to the antimicrotubule chemotherapy emtansine) versus trastuzumab alone as adjuvant therapy in patients with HER2-positive BC with residual disease after NAC. Results showed that the risk of recurrence of invasive BC or death was 50% lower with adjuvant trastuzumab and emtansine than with trastuzumab alone (HR 0.5, 95%CI [0.39–0.64], p<0.001) [33]. Results of the SWOG BR006 trial, which is currently in progress, are also expected. The study aims at assessing the impact of adjuvant pembrolizumab after surgery for 12 months on disease-free survival compared to observation in patients with TNBC and > 1 cm residual invasive cancer or positive lymph nodes after neoadjuvant chemotherapy [34]. Neoadjuvant trials are also warranted to determine the value of molecular and genetic markers to predict responsiveness to particular treatment regimens. As such, patients with little chance of responding to particular regimens could avoid the toxicity of futile therapy, and be study participants in evaluations of novel treatment strategies.
